# Two-step versus Single Application of Mitomycin-C in Photorefractive Keratectomy for High Myopia

**Published:** 2012-01

**Authors:** Farhad Fazel, Leila Roshani, Leila Rezaei

**Affiliations:** Eye Research Center, Feiz Hospital, Isfahan University of Medical Sciences, Isfahan, Iran

**Keywords:** Mitomycin C, Photorefractive Keratectomy, High Myopia

## Abstract

**Purpose:**

To evaluate the long-term outcomes of two-step versus single application of mitomycin-C (MMC) during photorefractive keratectomy (PRK) for high myopia.

**Methods:**

This randomized clinical trial included consecutive patients with high myopia (exceeding 7 D). Patients underwent PRK and were randomized to two methods of MMC 0.02% application as follows: in the single application group, MMC was applied for 45 seconds followed by irrigation; in the two-step group MMC was used identically followed by repeat application for another 15 seconds and corneal surface irrigation. Visual acuity, refractive error, pachymetry, topography, corneal haze and complications were compared between the two groups 18 months after surgery.

**Results:**

One hundred and forty patients (70 subjects in either study arm) underwent PRK according to the study protocol. Mean spherical equivalent refractive error was significantly reduced from baseline to −1.16±0.39 D in the single application group and to −1.07±0.39 D in the two-step group. Sixteen (11.5%) versus 8 (5.7%) eyes lost one or more line(s) of best corrected visual acuity in the single application group as compared to the two-step group (P=0.05). Corneal haze was observed in 18 (12.9%) and 8 (5.7%) eyes in the single application versus two-step group, respectively (P=0.04). Grade 3 corneal haze was not observed in the two-step group but occurred in five eyes (3.6%) in the single application group (P=0.03). No eyes developed corneal ectasia during the follow-up period.

**Conclusion:**

Two-step intraoperative application of MMC 0.02% in highly myopic eyes undergoing PRK can reduce the frequency and severity of haze formation.

## INTRODUCTION

Excimer laser photorefractive keratectomy (PRK) is a popular surgical procedure for correction of refractive errors.^1^–^6^ However, due to its side effects, i.e. slow visual recovery, discomfort in the early postoperative period and corneal haze, laser in situ keratomileusis (LASIK) remains an attractive alternative.^7^ Patients with myopia exceeding 5 diopters (D) and corneal thickness less than 500 μm are not suitable candidates for LASIK or conventional PRK due to inadequate corneal thickness and the risk of haze formation.^8^–^11^ In these eyes, LASIK may induce structural instability, irregular astigmatism, loss of best-corrected visual acuity (BCVA) and iatrogenic keratoconus.^12^

In highly myopic eyes unsuitable for laser ablation, phakic intraocular lens (PIOL) implantation may be a reasonable alternative. Highly myopic eyes may be treated by PRK if wound healing reactions such as haze formation could be effectively suppressed. PRK is an easy and non-invasive procedure as compared to PIOL implantation and avoids potential complications associated with intraocular surgery such as endophthalmitis. With very high amounts of myopia, PIOL implantation may be superior to PRK because of the considerable risk of haze formation.^13^,^14^

Corneal haze formation after PRK is induced by activation and proliferation of keratocytes. Collagen produced by activated keratocytes is much less organized than normal stromal collagen and shows matrix-free areas and fibers with irregular stereospatial relationship.^15^–^20^ Small case series have suggested that topical application of anti-proliferative agents such as mitomycin-C (MMC) can modulate haze formation and treat established haze.^21^–^25^ It has also been demonstrated that MMC application prevents corneal haze formation following PRK in moderate to high myopia.^18^–^21^,^26^–^29^

This study was designed to compare BCVA and corneal status 18 months after PRK employing two different methods of MMC 0.02% application, and to investigate long-term refractive and mechanical stability, and late-onset sequelae such as postoperative corneal haze.

## METHODS

This randomized clinical trial consecutively enrolled patients with high myopia (greater than −7 D). All patients underwent PRK with MMC application at Feiz Hospital, Isfahan, Iran. All subjects attended lectures about the procedure, were counselled before randomization and provided informed consent for the procedure. One skilled surgeon with extensive experience with PRK operated all eligible patients in group A (single application) and group B (two-step application) of MMC. All patients underwent a detailed ocular examination including subjective and cycloplegic refraction, keratometry, corneal pachymetry, tonometry and dilated fundus examination.

Candidates 20 to 50 years of age, with stable refractive error for at least 2 years, cycloplegic refractive error exceeding −7 D, spherical equivalent refraction of −7.00 to −10.00 D, regular astigmatism, BCVA of 0.2 logMAR or better, corneal thickness of 500 to 700 μm, keratometry values of 43 to 47 diopters and normal endothelial cell counts were included. For being eligible for PRK, residual corneal thickness (excluding the epithelium) was required to be greater than 350 μm. Patients with a history of ocular diseases or severe ocular trauma, previous keratorefractive procedures, contact lens wear during the past 2 months, wound healing abnormalities (e.g. keloids), systemic disorders (diabetes mellitus, systemic lupus erythematosus or other connective tissue disorders), anterior or posterior uveitis, ectatic disorders such as keratoconus, corneal dystrophies or degenerations, glaucoma, retinal diseases, and lens opacities were excluded.

### Operative Procedure

All patients received topical anesthesia without systemic sedation. Using an 8 mm ring, ethanol 16% was applied in a marker well for 20 seconds. The epithelium was then removed mechanically using a sponge. Conventional ablation was performed using the Nidek EC 5000 excimer laser machine with a central optical zone of 6 mm and transition zone of 7.5 mm. MMC 0.02% (0.2 mg/ml diluted in balanced salt solution) was applied immediately after laser ablation as follows: in the single application group, MMC was applied over the ablated stroma using a saturated sponge for 45 seconds. The corneal surface and the entire conjunctiva were then irrigated with 20–40 ml of cold normal saline solution. In the two-step group, MMC was applied for 45 seconds followed by irrigation as mentioned above. Thereafter, MMC was reapplied for another 15 seconds using the same concentration and in a similar fashion followed by corneal surface irrigation. At the end of the procedure, a bandage contact lens was applied which was removed after one week.

Postoperatively, patients were examined 1 and 3 days, 1 and 4 weeks, and 3, 6, 12 and 18 months after PRK. All subjects were instructed to take an oral analgesic (diclofenac sodium) every 8 hours, betamethasone eye drops every 4 hours for 2 weeks, and chloramphenicol eye drops every 6 hours for one week. During weeks 3 and 4, all patients were treated with betamethasone eye drops and artificial tears every 6 hours which were tapered to every 8 and 12 hours during the second and third postoperative months respectively and discontinued thereafter. All patients were instructed to wear sunglasses in direct sunlight for 3 months. During the first postoperative week, all patients underwent slit lamp biomicroscopy and the size of epithelial defect was measured using its ruler to identify time for complete re-epithelialization. At each visit, refraction and slit lamp examination were performed. At final follow-up (18 months), pachymetry and topography (Zeiss-Humphrey, Jena, Germany) were also obtained. We employed Hanna’s grading scale from 0 (no haze) to +4 (dense white corneal haze) to categorize corneal haze.^17^

Data analysis was performed using the SPSS software version 16. Refractive errors in eyes which required cylindrical corrections were converted to spherical equivalents (SE). Data of right and left eyes were analyzed independently as surgery was conducted independently for fellow eyes. The statistical level of significance was set at 0.05.

## RESULTS

Overall, 280 eyes of 140 patients including 70 subjects in either study arm were operated. The single application group consisted of 46 female (65.5%) and 24 male (34.3%) subjects and the two-step group included 50 female (71.3%) and 20 male (28.6%) patients (P=0.031). Mean patient age at the time of allocation was 26.3±2.6 (range, 20–46) years. Mean age was 25±3.7 years in the single application group and 25±4.2 years in the two-step group (P=0.070). Mean SE refractive error was −8.40±0.80 D in the single application versus −8.51±0.92 D in the two-step groups, respectively. No significant difference was found between the two groups in terms of preoperative manifest refraction and postoperative refractive error. SE was significantly (P=0.001) reduced to −1.16±0.39 D and −1.07±0.39 D in the single application and two-step groups, respectively. Table 1 summarizes changes in refractive parameters; mean change in SE are shown in Table 2.

BCVA did not change or was improved in 124 (88.5%) of eyes in the single application group and in 132 (94.3%) of eyes in the two-step group (P=0.05) (Table 3).

**Figure 1.**
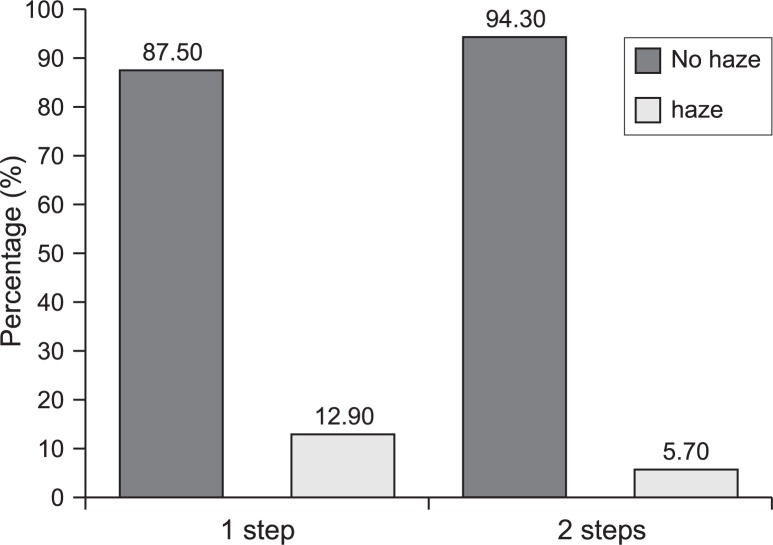
Percentage of haze formation in the study groups

After 18 months, corneal haze formation occurred in 18 (12.9%) of eyes in the single application group versus 8 (5.7%) of eyes in the two-step group (P=0.04). Grade 3 corneal haze was not seen in any eye in the two-step group but occurred in five eyes (3.6%) in the single application group (Table 4 and Fig. 1). Haze formation was noted in all patients with loss of BCVA.

There were no cases of progressive corneal ectasia up to 18 months follow-up. No complications such as eccentric ablation, delayed re-epithelialization, persistent epithelial defect, or microbial keratitis were observed.

## DISCUSSION

Treatment of high myopia using PRK remains a challenge. The higher rate of haze formation together with regression of refractive correction has decreased interest in PRK, as compared to LASIK, for treatment of high ametropias, including high myopia.^7^,^18^,^26^,^30^ However, LASIK may not be feasible in eyes with insufficient residual corneal stromal thickness. One solution is application of smaller ablation zones; this may lead to impaired night vision, halos, blurred vision, and ghost images.

Recently, the role of PRK has been reappraised because of corneal instability and poor visual function frequently reported in highly myopic patients following LASIK.^7^,^31^ Nevertheless, haze formation continues to be the major drawback to PRK for high mypoia.^32^ If the safety and predictability of PRK with MMC can be verified, larger ablation depths may be employed and the above mentioned complications will be avoided, making surface ablation a safe alternative for correction of high refractive errors.^33^

MMC is an antimetabolite used in ophthalmology for cases of corneal and conjunctival intraepithelial neoplasms, ocular pemphigoid and during surgical treatment of glaucoma and pterygium. It employs its cytotoxic effects through inhibition of DNA synthesis. The rationale for its use in PRK is based on prevention of keratocyte proliferation and deposition of irregularly generated material leading to scar formation.^34^,^35^ The effect of MMC 0.02% in preventing haze formation has previously been shown by Talamo et al^36^ and Xu et al^37^ in experimental models. It has also been reported that the application of MMC can decrease pre-existing haze following PRK and radial keratectomy.^21^–^23^ Carones et al^18^ reported acceptable refractive and visual outcomes over a 6-month period following PRK with MMC 0.02% for moderate and high myopia.

Kymionis et al^38^ evaluated aqueous humor concentrations of MMC following PRK in 24 eyes of 12 male rabbits in 4 groups. Eyes in groups 1 and 2 underwent PRK to correct 5 D of myopia in a 6-mm optical zone followed by MMC 0.02% application on the ablated corneal stroma for 60 and 120 seconds, respectively. Eyes in groups 3 and 4 underwent PRK to correct 10 D of myopia in a 6-mm optical zone followed by MMC 0.02% application for 60 and 120 seconds, respectively. Mean aqueous humor concentrations of MMC were 0.23±0.03, 0.39±0.05, 0.28±0.04 and 0.52±0.16 μg/mL in groups 1, 2, 3, and 4, respectively. The investigators showed that both duration of exposure (P<0.001) and ablation depth (P=0.019) were significantly correlated with MMC concentration in the aqueous humor.

Haze formation and regression after PRK are more common in people of Middle Eastern origin.^39^ Other important predictors of haze formation are the amount of correction and the size of ablation zone. A 6 mm optical zone and a minimum of 7 D of correction, practically limited our study to patients who were at a high risk of haze formation following PRK. Maximum ablation depth in our patients was 160 μm, minimal residual corneal thickness was 400μm and no patient developed corneal ectasia after 18 months. It seems that residual corneal thickness of 400 μm after PRK is safe in terms of preventing iatrogenic ectasia, however further studies are warranted to approve this conclusion.

The current study was designed to evaluate the efficacy and long-term stability of PRK with single (45 seconds) versus two-step (45 seconds followed by 15 seconds) application of MMC 0.02% on the ablated stromal bed in highly myopic eyes. We sought to determine whether a small increase in duration of MMC application can decrease corneal haze formation following PRK for high myopia. To decrease penetration of MMC into the corneal stroma and anterior chamber, we irrigated the corneal surface after MMC application.

Refractive and visual outcomes in our series were better than studies on LASIK for correcting the same amount of myopia.^40^,^41^ Moreover, outcomes of two-step application of MMC were better than those of a single application (routine practice). We found no similar study to compare our results with. Out of 83 articles investigating MMC administration for PRK in the PubMed database, only a few compared different durations of MMC application.

Wallau et al^42^ reported the one-year results of PRK with one minute application of MMC for customized correction of myopia. Clinically significant haze was not found following surface ablation; 74% of MMC-PRK and 43% of LASIK eyes gained one or more lines of BCVA 12 months postoperatively. Thornton et al^43^ compared the safety and efficacy of low concentration MMC (0.002%) with that of standard concentration (0.02%) in eyes undergoing PRK for myopia. The duration of exposure varied from 30 seconds to 2 minutes in both groups. With multivariate analysis, significantly less haze formation was noted at all postoperative time points with the standard dose in eyes with high myopia and greater ablation depths. In contrast, haze levels were similar with both MMC concentrations in eyes with moderate myopia and lower ablation depths. In the subset of contralateral eyes which randomly received low dose MMC (0.002%) for 30 seconds or 2 minutes, no significant difference was found in terms of haze formation.

Considering the fact that haze influences BCVA, we independently analyzed BCVA in our study. With a single application of MMC, 16 eyes (11.5%) lost one or more line(s) of BCVA after surgery as compared to 8 eyes (5.7%) in the two-step MMC group. Major side effects (corneal edema, melting or perforation) or minor complications (corneal sensitivity or tear film problems) associated with the use of MMC were not observed in any of the study groups. None of the eyes in the two-step group had significant grades of corneal haze or degenerative reactions after surgery. Many studies have highlighted the toxicity of MMC on the corneal endothelium, however our study was not designed to address this issue. Considering the corneal penetration of MMC and susceptibility of its endothelium, additional studies are needed to determine the long-term impact of MMC use during PRK on the corneal endothelium.^44^ Another important finding in our study was the absence of corneal ectasia despite increased attempted corrections. Corneal ectasia following refractive surgery is a long-term complication which may appear up to a decade after initial treatment, therefore, longer follow-up is necessary to draw a definite conclusion.

Refractive surgery procedures such as PRK which involve mechanical removal of the corneal epithelial barrier, excimer laser stromal ablation, and subsequent MMC treatment, expose the cornea to substantial levels of MMC. Length of exposure and MMC concentration in the endothelium and aqueous humor are directly related to the amount of MMC applied to the anterior surface. Based on established mechanisms of MMC action, *in vitro* and animal studies, and reports of intraocular penetration after MMC in non-PRK treatments, MMC has the potential to damage cellular DNA.^45^,^46^ However, corneal irrigation between stepwise MMC application can theoretically reduce the risk of these complications.

In conclusion, longer duration of MMC administration, i.e. 45 seconds followed by an additional 15 seconds, in PRK for myopia higher than −7 D seems to be safe and may reduce corneal haze formation after surgery.

## Figures and Tables

**Table 1.** Refractive data for 280 eyes of 140 patients

**Parameter**	**Mean ± SD (range)**	
	**Preoperative**	**Postoperative**
CIM	1.09 ± 0.74 (0.46 to −5.55)	1.93 ± 10.14 (0.18 to −1.16)
SF	0.347 ± 0.182 (−0.66 to 0.62)	−0.951 ± 0.622 (−1.23 to 1.33)
Pachymetry (μm)	532 ± 37 (481 to 643)	432 ± 38 (390 to 553)

SD, standard deviation; CIM, corneal irregularity measure; SF, shape factor

**Table 2.** Changes in spherical equivalent refractive error in either study group

	**Single application**		**Two-step**		P-value*
	**Mean ± SD**	**Min, Max**	**Mean ± SD**	**Min, Max**	
Pre-op	−8.4 ± 0.8	−7.25, −10	−8.57 ± 0.92	−7.2, −10.75	0.08
Post-op	−1.16 ± 0.39	−0.50, −2.00	−1.07 ± 0.39	−0.25, −1.75	0.06

SD, standard deviation

* Independent *t*-test

**Table 3.** Changes in best-corrected visual acuity in the study groups

	**Lines lost**		**Unchanged**	**Lines gained**	
	≤ **2**	**1**		**1**	≤ **2**
Single application	5 (3.6%)	11 (7.2%)	112 (80%)	10 (7.1%)	2 (1.4%)
Two-step	2 (1.4%)	6 (4.3%)	114 (81.4%)	12 (8.6%)	6 (4.3%)

*t*-test, P=0.05

**Table 4.** Corneal haze formation at final visit

**Corneal haze scale**	**Single application**		**Two-step**	
	**N**	**%**	**N**	**%**
0	122	1.87	132	3.94
+1	7	5	6	3.4
+2	6	3.4	2	4.1
+3	5	6.3	0	0
Total	140	100	140	100
